# Selective immunoglobulin A deficiency in children with diabetes mellitus: Data from a medical center in Ukraine

**DOI:** 10.1371/journal.pone.0277273

**Published:** 2022-11-17

**Authors:** Oksana Boyarchuk, Lesya Dobrovolska, Halyna Svystunovych

**Affiliations:** I.Horbachevsky Ternopil National Medical University, Ternopil, Ukraine; Sohag University Faculty of Medicine, EGYPT

## Abstract

The aim of this study was to estimate the prevalence of selective immunoglobulin A deficiency (SIgAD) among children with type 1 diabetes mellitus (DM) in Ternopil region (western Ukraine). Serum IgA levels were measured in 240 patients aged 4–17 years with DM and in 324 children of a control group of the same age. Normal IgA level was observed in 210 (87.5%) patients, increased—in 18 (7.5%), decreased (lower than the age reference value)—in 12 (5.0%) patients with DM. The mean IgA level in patients with DM was 152.11±73.78 mg/dL. SIgAD criteria were met by 7 (2.9%) children with DM, but none of the children of the control group met the SIgAD criteria. Female / male ratio among the patients with SIgAD was 1/6. There was no history of recurrent infections in these patients. No correlation between IgA and HbA1c levels was detected. Autoimmune thyroiditis was observed in 42.9% of patients with DM and SIgAD, and in 3.5% of patients with DM and normal or increased IgA levels. Thus, the prevalence of selective IgA deficiency in children with DM in Ternopil region (Ukraine) is 2.9% (1:34). This study shows that patients with low IgA levels need further re-examination of IgA levels to exclude SIgAD. Children with SIgAD and DM should be monitored for autoimmune manifestations that may affect the course and consequences of the disease.

## Introduction

Selective immunoglobulin A deficiency (SIgAD) is one of the most common primary immunodeficiencies (PID) [1]. Its prevalence ranges from 1:300 to 1:3000 depending on a population [1, 2]. SIgAD is diagnosed in children older than four years with serum IgA levels below 7 mg/dL, with normal immunoglobulins G (IgG) and M (IgM) and other causes of hypogammaglobulinaemia and T cell defects ruled out [1, 3].

SIgAD is a heterogeneous condition with multifactorial mechanisms and its pathogenesis is not completely understood [1]. Typically, IgA deficiency is associated with a defect in B cells failing to produce IgA [3]. T cell abnormalities and cytokine dysregulation may also play a role [4].

The clinical picture of SIgAD varies from an asymptomatic course, when IgA deficiency is identified accidentally, to a course with symptoms that may be similar to that of other congenital immune defects, including recurrent infections, autoimmune diseases, allergic manifestations and malignancy [5–8]. The most common clinical manifestations of SIgAD are respiratory and gastrointestinal infections, followed by allergy and autoimmune disorders [6, 9]. There is a possibility of a shared genetic background in SIgAD and autoimmunity, considering familial clustering of these diseases and the link of HLA 8.1 haplotype to different autoimmune conditions [9]. Children with SIgAD are at higher risk of developing type 1 diabetes mellitus, juvenile idiopathic arthritis, rheumatoid arthritis, ankylosing spondylitis, systemic lupus erythematosus, hyperthyroidism, hypothyroidism, Crohn’s disease, ulcerative colitis, and vitiligo [10, 11]. Early diagnosis of SIgAD can help to timely detect autoimmune diseases and to prevent the development of infectious complications in this group of patients.

An increase in the incidence of SIgAD in patients with type 1 diabetes mellitus (DM) has been shown in several publications [10, 12, 13], although the prevalence and other clinical manifestations of SIgAD vary depending on a population. The prevalence of IgA deficiency in patients with type 1 diabetes in Ukraine has not been studied. Determining the prevalence of SIgAD among children with type 1 DM will allow us to understand the scope of this disease and the need to monitor for other manifestations of immunodeficiency that may affect the health of these patients.

The aim of this study was to estimate the prevalence of SIgAD among children with type 1 diabetes mellitus in Ternopil region (western Ukraine).

## Materials and methods

This cross-sectional study involved 240 children aged 4–17 years with DM who were referred to the Endocrinology Unit of Ternopil Regional Children’s Hospital from March 2019 to November 2021. All patients with DM that were admitted to the Endocrinology Unit during this period were enrolled. A thorough analysis of clinical and laboratory parameters in the medical records of all patients was performed. The control group comprised 324 children of the Outpatient Department of Ternopil Regional Children’s Hospital that were admitted during the same period (from March 2019 to November 2021). Exclusion criteria for the control group were PID, acute period of respiratory diseases and gastroenteritis, autoimmune and allergic diseases. None of the patients with DM and of the control group received immunoglobulin replacement therapy or immunosuppressive therapy.

Serum IgA was measured in all patients using an immunoturbidimetric assay. If reduced IgA levels of <7 mg/dL were detected, the test was repeated.

For all patients with IgA levels below the reference value, face-to-face interviews were conducted with children and their parents aiming to identify the symptoms of PID, autoimmune and non-autoimmune comorbidities, and a detailed family history taken.

If IgA deficiency was detected, serum IgM, IgG, IgE; IgG subclasses (IgG1, IgG2, IgG3, IgG4); lymphocytes subclasses and antibody response to vaccinations were measured to rule out other immunodeficiencies. ELISA was used to determine specific immunoglobulin G antibodies against diphtheria and tetanus, and IgG subclasses. Immunoturbidimetric assay was used to measure serum immunoglobulins M and G, solid-phase chemiluminescent immunoassay was used for IgE detection; and flow cytometry was used to measure lymphocytes subclasses.

We also determined antibodies IgA and IgG to gliadin (AGA) and to tissue transglutaminase (anti-tTG), and antibodies to thyroid peroxidase (ATPO).

Diagnosis of SIgAD was based on the criteria of the European Society for Immunodeficiencies (ESID) [14]. According to these criteria, SIgAD is diagnosed in patients older than 4 years of age with IgA value less than 7 mg/dL, but normal serum IgG and IgM (measured at least twice), normal IgG antibody response to vaccinations and with secondary causes of hypogammaglobulinaemia and T-cell defects ruled out.

The study was performed following the tenets of the 1975 (revised in 2000) WMA Declaration of Helsinki; it was approved by the I.Horbachevsky Ternopil National Medical University Ethics Committee (Protocol № 51 of 14 January 2019). A written informed consent was obtained from the parents of all study participants.

Statistical analysis was carried out using the statistical package STATISTICA 10.0 and table editor Microsoft Excel 2003. Continuous variables were expressed as a mean and standard deviation (SD). The comparison of frequency parameters was performed using the Chi-square test. Odds ratio (OR) and its 95% confidence interval (95% CI) were used to measure associations between SIgAD and the incidence of other diseases in patients with DM. Spearman’s correlation coefficient was used to determine the rank measure of association. The differences between the parameters were accepted as statistically significant at p<0.05.

## Results

Among the 240 children with type 1 DM enrolled in the study, normal IgA was observed in 210 (87.5%), elevated in 18 (7.5%), and reduced (lower than the age reference value)—in 12 (5.0%) patients. Among the 12 patients with low IgA, 7 (2.9%) children met the SIgAD criteria. Among the 324 children of the control group, normal IgA was observed in 291 (89.8%), elevated in 5 (1.5%), and reduced in 28 (8.6%). None of the patients in the control group met the SIgAD criteria. The lowest level of IgA in this group was 27 mg/dL.

To identify the impact of SIgAD on the diabetes course, the patients were divided into three groups: those with normal and high IgA; with low IgA (but >7 mg/dL); and diagnosed with SIgAD. There was no significant difference among the groups by the patient age (Table 1).

**Table 1:** Baseline characteristics of the patients with DM.

Characteristic	DM + normal or increased IgA	DM + decreased IgA	DM + SIgAD	All DM patients
	n = 228	n = 5	n = 7	240
Male, n (%)	118 (51.8)	3 (60.0)	6 (85.7)	127 (52.9)
Female, n (%)	110 (48.3)	2 (40.0)	1 (14.3)	113 (47.1)
Age at clinic visit, yr (mean±SD)	11.11±3.71	12.20±3.25	10.43±2.57	11.12±3.67
Age at DM onset, yr (mean±SD)	7.16±3.57	7.60±4.16	7.57±2.51	7.18±3.55
DM duration, yr (mean±SD)	3.95±3.46	4.30±4.09	3.02±2.14	3.92±3.43
Ketoacidosis at DM onset, n (%)	97 (42.5)	2 (40.0)	4 (57.1)	103 (42.9)
HbA1c, % (mean±SD)	8.73±2.37	7.06±0.37	9.16±2.10	8.71±2.36
HbA1c <7%, n (%)	66 (28.9)	3 (60.0)	1 (14.3)	70 (29.2)
C-peptide, ng/ml (mean±SD)	0.31±0.23	0.24±0.08	0.32±0.22	0.31±0.22
IgA, mg/dL (mean±SD)	158.56±69.09	46.02±22.66	2.49±2.79	152.11±73.78
**Comorbidities**, n (%)				
Goiter	16 (7.0)	0	0	16 (6.7)
Autoimmune thyroiditis	8 (3.5)	0	3 (42.9)	11 (4.6)
Hypothyroidism	6 (2.6)	0	1 (14.3)	7 (2.9)
Celiac disease	4/108 (3.7)	0	0	4/120 (3.3)
Arthritis	1 (0.4)	0	0	1 (0.4)
Psoriasis	1 (0.4)	0	0	1 (0.4)
Atopic dermatitis	1 (0.4)	0	0	1 (0.4)
Organomegaly	9 (3.9)	0	1 (14.3)	10 (4.2)
(hepatomegaly, splenomegaly, lymphadenopathy)				
**DM complications**, n (%)				
Lipohypertrophy	16 (7.0)	0	1 (14.3)	17 (7.1)
Mauriac syndrome	1 (0.4)	0	0	1 (0.4)
Diabetic neuropathy	2 (0.9)	0	0	2 (0.8)

HbA1c, glycosylated hemoglobin

Overall, there was no difference among all participants by gender, with a slightly higher number of boys (127, 52.9%) (Table 1). However, in the group of children with SIgAD and DM, boys predominated (6/7). None of the seven patients with SIgAD had family history of primary immunodeficiency, early death and consanguineous parents. Among patients of the control group, 171 (52.8%) were female, the mean age was 11.24±3.51 years. The mean IgA level in the control group was 146.71±49.35 mg/dL.

The mean age of DM onset was 7.18 ± 3.55 years, with no difference between the groups (Table 1). In 2 children DM manifested before the age of one year, and in 63 (26.3%) patients—after 10 years of age. It should be noted that in 103 (42.9%) patients, the newly diagnosed diabetes was accompanied by ketoacidosis.

The mean duration of DM at the time of IgA determination was 4.34 ± 3.35 years. Glycosylated hemoglobin (HbA1c) ranged from 5.2% to 15.8% and averaged 8.71 ± 2.36%, with no significant difference between the groups (p>0.05). Only 70 (29.2%) patients reached the target level of HbA1c <7% recommended by the American Diabetes Association [15], and in the group of children with SIgAD diabetes this level was reached in only 1 patient. We did not find any correlation between IgA levels and HbA1c.

At the same time, the examined patients had other conditions, among which the most prevalent ones were thyroid diseases: goiter (6.7%), autoimmune thyroiditis (4.6%), and hypothyroidism (2.9%) (Table 1). In the patients with DM and SIgAD we observed a significantly higher rate of autoimmune thyroiditis compared to the patients with DM and normal or elevated IgA levels (42.9% versus 3.5%; OR = 20.6; 95% CI: 3.94 to 107.92; p = 0.0003). Other comorbid conditions have occurred in several cases (celiac disease in 4 (3.3%) patients; arthritis, psoriasis, atopic dermatitis–each in 1 case (0.4%). Organomegaly (hepatomegaly, splenomegaly, lymphadenopathy) occurred in 4.2% of the patients with DM. Hepatosplenomegaly was observed in one patient, and hepatomegaly in the other patients. Among the complications, lipohypertrophy was the most common (7.1%).

The main clinical and laboratory parameters in patients with DM and SIgAD are presented in Tables 2 and 3. These findings have confirmed SIgAD diagnoses, since all other immunological parameters, including IgM, IgG, IgG subclasses, lymphocyte subpopulations, and IgG antibody response to vaccines were within normal limits.

**Table 2:** The main laboratory findings in patients with DM and SIgAD.

Parameter	Mean±SD	Reference range
IgA, mg/dL	2.49±2.79	22–274
IgM, mg/dL	101.85±32.26	13–145
IgG, mg/dL	1414.07±353.59	654–1600
IgE, IU/ml	208.65±199.89	0–80
IgG1, mg/dL	823.00±150.80	342–1150
IgG2, mg/dL	511.57±272.82	76–455
IgG3, mg/dL	71.29±27.64	17–173
IgG4, mg/dL	11.61±7.66	1.6–136
CD3, %	65.54±8.56	66–76
CD3, cells/m^3^	1612.57±342.80	1400–2000
CD4, %	31.72±6.02	33–41
CD4, cells/m^3^	777.85±190.44	700–1100
CD8, %	29.21±6.26	27–35
CD8, cells/m^3^	725.57±245.76	600–900
CD19, %	12.12±4.69	12–22
CD19, cells/m^3^	309.14±157.42	300–500
CD4/CD8	1.12±0.31	1.1–1.4
Anti-diphtheria antibodies, IU/ml	0.42±0.37	>0,1
Anti-tetanus antibodies, IU/ml	1.42±1.52	>0,1
ATPO, IU/ml	142.81±278.94	0–30
AGA-IgA, IU/ml	5.84±6.49	<40–6–12 yr; <25—after12 yr.
AGA-IgG, IU/ml	18.98±19.51	<35–6–12 yr; <25—after12 yr.
anti-tTG -IgA, IU/ml	0.27±0.24	0–20
anti-tTG -IgG, IU/ml	10.47±8.18	0–25

ATPO, antithyroidperoxidase antibodies; AGA, anti-gliadin antibodies; anti-tTG, antibodies to tissue transglutaminase.

**Table 3:** Clinical and laboratory data of patients with DM and SIgAD.

Parameter	Patient 1	Patient 2	Patient 3	Patient 4	Patient 5	Patient 6	Patient 7
Age, yr	14	10	8	9	8	14	10
Gender	M	M	M	M	F	M	M
DM duration, yr	4.5	3.8	0.1	5.7	3.5	0.1	3.5
Ketoacidosis at DM onset	Yes	No	Yes	No	No	Yes	Yes
HbA1c, %	9.9	8.4	13.3	8.7	9.4	6.9	7.6
C-peptide, ng/ml	0.1	na	0.2	na	0.22	0.4	0.67
IgA, mg/dL	<0.8	<0.4	<0.5	<0.3	<7	2.7	5.7
IgM, mg/dL	84	148	80	72	128	71	130
IgG, mg/dL	1234	1743	907	1220	1975	1403	1417
IgE, IU/ml	na	na	na	350	67.3	na	na
IgG1, mg/dL	812	924	720	587	975	998	745
IgG2, mg/dL	423	682	91	562	894	265	664
IgG3, mg/dL	39	92.4	74	95	85.8	87	25.8
IgG4, mg/dL	21.1	23.0	2.4	8.5	11.8	7.3	7.2
CD3, %	49,1	61.2	71.2	67.3	72	64	74
CD3, cells/m^3^	957	1903	1680	1870	1530	1444	1904
CD4, %	22,1	25	37	37	31	36	34
CD4, cells/m^3^	430	777	863	1030	655	816	874
CD8, %	21	39	28	23.5	35	27.5	30.5
CD8, cells/m^3^	409	1212	660	650	744	618	786
CD19, %	8,4	14	10	22	11	9.5	10
CD19, cells/m^3^	163	435	245	610	235	213	263
CD4/CD8	1,05	0.64	1.3	1.6	0.88	1.3	1.1
Anti-diphtheria antibodies, IU/ml	0.450	0.190	0.304	1.175	0.192	0.104	0.552
Anti-tetanus antibodies, IU/ml	3.354	0.572	0.612	3.861	0.201	0.419	0.937
ATPO, IU/ml	179.16	43.27	2.78	11.8	1.39	2.58	759
AGA-IgA, IU/ml	0.28	12.42	3.51	2.74	3.97	0.60	17.34
AGA-IgG, IU/ml	0.51	50.24	1.32	11.32	23.98	6.29	39.21
anti-tTG-IgA, IU/ml	0.07	0.08	0.58	0.07	0.54	0.09	0.47
anti-tTG-IgG, IU/ml	14.91	17.37	1.47	4.46	23.07	9.10	2.93
Autoimmune thyroiditis	Yes	Yes	No	No	No	No	Yes
Hypothyroidism	No	No	No	No	No	No	Yes
Gluten intolerance	No	Yes	No	No	No	No	Yes

HbA1c, glycosylated hemoglobin; ATPO, antithyroidperoxidase antibodies; AGA, anti-gliadin antibodies; anti-tTG, antibodies to tissue transglutaminase; na, not available.

The patient 3 with SIgAD (Table 3) experienced a decrease in IgA levels (<0.5 mg/dL) twice since DM was first diagnosed. After administration of insulin therapy, his IgA level has increased, but was still below the reference range. In the patient 7, at the first examination when the duration of diabetes was 1 year, low IgA level (18.2 mg/dL) was detected, while in two subsequent examinations, the IgA levels were less than 7 mg/dL, which allowed referring him to the group of patients with SIgAD.

AGA-IgG were elevated in two patients with DM and SIgAD, although anti-tTG-IgG was normal (Table 3).

## Discussion

Changes in IgA levels in patients with DM have been described previously [16]. The researchers reported an increase not only in IgA levels in the patients with type 1 and type 2 DM compared to healthy people [16], but also changes of other immunoglobulins, which varied depending on the age and duration of the disease [17]. In our cohort of children with DM, we observed an increase in IgA levels above the reference range in 7.5% of children.

Even though there is a number of publications on the prevalence of SIgAD in children and adults with DM in countries of Europe and the world [10–13, 18], in Eastern Europe and in Ukraine in particular, such studies have not been conducted. Overall, the prevalence of SIgAD in children with type 1 DM was found to be 2.9% or 1:34, while there were no cases of SIgAD among the 324 children of control group. Our results are generally consistent with other studies that showed that the prevalence of SIgAD in patients with DM ranges from 0.38% (1:263) in adults in France [19] and 0.7% (1:143) in children in Iran [10] to 5.3% (1:19) in adults in western Sicily [13]; all these values exceed the prevalence of SIgAD in general populations (from 1:300 to 1:3000) [1, 9, 19]. Our study results were similar to those of a study in Greece, where the prevalence of SIgAD in children with type 1 DM was 3% [1].

Notably for this study, in our cohort we observed 5 children with low IgA levels, who nevertheless did not meet the ESID criteria of SIgAD [14]. However, we suggest that this group of patients also deserves attention in terms of on-going monitoring. The results of examinations of the patients 3 and 7 showed that their IgA levels ranged from below the reference values, but over 7 mg/dL to less than 7 mg/dL. Changes in immunoglobulin levels after insulin therapy have been reported in another study [20]. These results indicate the need for a repeated assessment of IgA levels in the patients with newly diagnosed diabetes and monitoring these levels after insulin therapy administration. Therefore, the group of patients with reduced IgA levels, despite these levels being over 7 mg/dL, needs further monitoring to exclude SIgAD.

Boys predominated (85.7%) in our cohort of patients with SIgAD and DM. These findings are consistent with the results of studies from Italy and Iran, where boys also predominated (87.5% and 100.0%, respectively) [10, 13]. However, the study from Greece did not show a significant sex difference in SIgAD prevalence [12].

We also found no correlation between IgA and HbA1c levels, which is consistent with other studies [10, 12, 13, 21], although there are some reports of inverse correlation between IgA and HbA1c [18]. One study reported a correlation between salivary IgA and HbA1c level [22]. In the group of patients with SIgAD, the target level of HbA1c <7% was achieved in only 1 (14.3%) patient, while the average in all patients was twice as high (29.2%). This is lower than in a large population-level study where 20–25% of children and adolescents had reached the target levels of HbA1c [20].

We did not observe a higher incidence of recurrent infections among children with SIgAD, which corresponds to the results of other studies [12, 13]. However, it should be noted that among comorbid conditions, we detected a high frequency of other autoimmune diseases, including autoimmune thyroiditis (42.9%) in patients with DM and SIgAD, similarly to other studies which showed a higher prevalence of autoimmune thyroiditis in this cohort of patients, reaching the frequency of 62 to 80% [13, 21]. The prevalence of autoimmune thyroiditis among children with DM with normal IgA level in various studies was 11.3% [23], 14.4% [24] to 27.1% [21] depending on the age and duration of DM. In adults, this frequency ranged from 28.9% [13] to 35.4% [25]. Our results showed a slightly lower prevalence of autoimmune thyroiditis in children with DM only, which may be correlated with their age and the duration of DM, but the trend of its higher frequency in SIgAD patients persisted.

Children with primary immunodeficiency, including SIgAD, have an increased susceptibility to autoimmune diseases [11, 21, 26–28]. However, we did not confirm celiac disease in patients with DM and SIgAD, while two of them had gluten intolerance (high AGA-IgG, but normal anti-tTG-IgG). Our results are consistent with other studies [12, 13]. A number of associations have been reported between SIgAD and other autoimmune diseases, including type 1 diabetes mellitus, Graves’ disease, systemic lupus erythematosus, celiac disease, myasthenia gravis, and rheumatoid arthritis with the major histocompatibility complex region [27, 29]. This points out to genetic background of autoimmune diseases and genetic predisposition of the patients with SIgAD to DM and other autoimmune disorders. However, a limitation of this study is that there were no twins or relatives among the patients with autoimmunity, compared to other reports [29].

The need for screening for SIgAD in patients with DM is being discussed. An argument against it is the absence of classic symptoms of immunodeficiency, including recurrent respiratory, gastrointestinal and other infections, which could have affected the course and consequences of diabetes. However, it has been shown that patients with SIgAD are at an increased risk of death in the first 10 to 15 years after the diagnosis [30]. Therefore, most scientists agree on the need for IgA screening in patients with DM, which can help to conduct adequate monitoring and treatment, and might have positive consequences for the course of the disease [12, 13].

## Conclusions

The prevalence of selective IgA deficiency in children with DM in Ternopil region (Ukraine) is 2.9% (1:34), with its predominance in males (85.7%). Children with SIgAD and DM are at increased risk of autoimmune thyroiditis (42.9%). There was no history of recurrent infections in these patients. The study showed that patients with low IgA levels need further re-testing of IgA levels to exclude SIgAD. Children with SIgAD and DM should be monitored for autoimmune manifestations that may affect the course and consequences of the disease.

## Supporting information

S1 Data (XLSX)
